# Repeatability and Reproducibility Standard Deviations in the Measurement of Trace Moisture Generated Using Permeation Tubes

**DOI:** 10.6028/jres.108.022

**Published:** 2003-06-01

**Authors:** Peter H. Huang, Raghu Kacker

**Affiliations:** National Institute of Standards and Technology, Gaithersburg, MD 20899-0001, U.S.A.

**Keywords:** humidity standard, interlaboratory evaluations, measurement uncertainty, permeation-tube, repeatability, reproducibility, trace moisture measurement

## Abstract

Permeation-tube moisture generators are used in industry as calibrated sources of water vapor and carrier gas mixtures. Measurements were made using three permeation-tube moisture generators of the type used in the semiconductor industry. This paper describes repeatability and reproducibility standard deviations in measurement of moisture concentration from such generators. Repeatability refers to measurements within a system and reproducibility refers to measurements between systems. Two independent methods were used to measure the realized concentration of water vapor. The first measurement, the calculated value, was determined using calibrated permeation rate of permeation-tube and flow rate of dry carrier gas. This is the industrial method of evaluating moisture concentration. The second measurement, the measured value, was determined using the low frost-point generator at the National Institute of Standards and Technology (NIST) and a quartz-crystal-micro-balance. Four pairs of independent measurements for each generator and for six nominal levels in the range from10 nL/L to 100 nL/L were made. The characteristic used to quantify repeatability and reproducibility standard deviations in industrial measurements is the calculated value minus the measured value. Repeatability standard deviation ranges from 1 nL/L to 2 nL/L, approximately. Reproducibility standard deviation ranges from 2 nL/L to 8 nL/L, approximately. The documentary ASTM standard E691-99 was used for both data validation and quantification of the repeatability and reproducibility standard deviations.

## 1. Introduction

Permeation-tube moisture generators (PTMGs) were used to produce water vapor in a stream of pure nitrogen carrier gas. A PTMG produces a stable flow of water vapor permeating through a membrane-tube containing liquid water at a constant temperature and pressure. The water vapor is mixed with a metered stream of dry carrier gas. The concentration of moisture is calculated using the permeation rate of water vapor and the flow rate of dilution gas as discussed in Ref. [1]. This type of apparatus is commonly used in the semiconductor industry as a portable standard for the calibration of hygrometers.

Another apparatus for producing low levels of moisture in a stream of carrier gas is the low frost-point generator (LFPG) at the National Institute of Standards and Technology (NIST), which is based on thermodynamic principles. Compressed gas is purified and passed through a long-coiled saturator that is controlled at a stable temperature. The gas leaves the saturator completely saturated with water vapor in equilibrium at an absolute pressure and temperature. Measurements of the pressure and temperature within the saturator, along with the known relationship between the equilibrium water vapor pressure and the temperature of the ice in the saturator are used to determine the water vapor concentration. Additional details are given in Ref. [2].

The object of this study is to quantify the variation in measurement of water vapor generated by PTMGs used in industry. Two types of variation are of interest: variation within a system and variation between systems. The *Guide to the Expression of Uncertainty in Measurement* [3] defines repeatability conditions as the same conditions of measurement over a short period of time and reproducibility conditions as changed conditions of measurement. The change in this study is the use of different PTMG instruments. The terms repeatability standard deviation and reproducibility standard deviation as used here are contractions of “standard deviation in repeatability conditions” and “standard deviation in reproducibility conditions” respectively.

Data on the measurement of moisture content were collected over a period of 2 years for nominal water vapor concentrations of 10 nL/L, 20 nL/L, 40 nL/L, 60 nL/L, 80 nL/L, and 100 nL/L generated from three PTMGs. This range of concentration is important in the specification of gases such as nitrogen used in the semiconductor industry. The three PTMG instruments are labeled here as A, B, and C. The PTMGs were constructed on the same principle but with different hardware and represent typical PTMGs used in industry. They were loaned to NIST for measurement. The PTMGs were calibrated at the source, thus they represent industrial use conditions. The repeatability measurements for each instrument were made sequentially over a relatively short period of time. The reproducibility measurements using different instruments were made over a relatively long period of time. Thus the reproducibility represented here is a relatively long-term variation.

For each nominal level of moisture concentration, the actual concentration of water vapor produced by the PTMG was determined by two independent measurement methods. The two measurements are referred to here as the calculated value *x*_c_ and the measured value *x*_m_. The calculated value *x*_c_ was determined using the calibrated permeation rate of the permeation-tube and the calibrated flow rate of the dry carrier gas. The measured value *x*_m_ of moisture concentration was determined by a standard substitution method using the NIST's low frost-point generator (LFPG) and a quartz-crystal-micro-balance. We made four pairs of independent repeat measurements for each level and each generator.

In industry, the calculated value *x*_c_ is used as the amount of water vapor concentration produced by a PTMG. The measurand, quantity subject to measurement, is the actual moisture concentration. Repeatability and reproducibility standard deviations are defined for a fixed value of the measurand. In this application it is not practical to realize a fixed level of moisture. There is always some variation in the actual moisture concentration about the nominal level. So the characteristic used here to quantify repeatability and reproducibility standard deviations in industrial measurements is the difference δ*x* = *x*_c_–*x*_m_, where *x*_c_ and *x*_m_ are the calculated and measured values of the same actual moisture concentration determined simultaneously. This is a different scale for quantifying repeatability and reproducibility standard deviations. The measured values from the NIST LFPG are highly repeatable and they are in effect used as reference values to quantify repeatability and reproducibility standard deviations of PTMG measurements.

## 2. Statistical Analysis

**Fig. 1:**
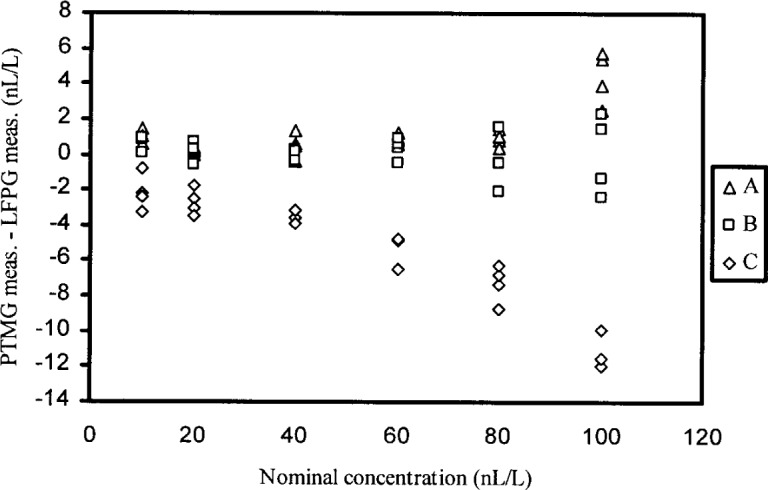
Difference of Permeation-tube moisture generator (PTMG) measurement called calculated value and the NIST low frost-point generator (LFPG) measurement called measured value. Some data points overlap.

The data on the difference (δ*x*) of calculated value *x*_c_ from measured value *x*_m_ are presented in Table 1 and plotted in Fig. 1. The statistical analysis has two objectives. First, the data are investigated to validate their suitability for quantifying repeatability and reproducibility standard deviations. Then repeatability and reproducibility standard deviations are quantified for validated data. We have used the statistical method recommended by ASTM standard E691-99 [4] to investigate the data as well as to quantify repeatability and reproducibility standard deviations. This documentary standard and its previous editions have existed for over 20 years. Despite being very useful, it does not seem to have been widely used. One of our objectives is to show its utility. The statistical analysis is done separately for each level of nominal concentration.

**Fig. 2:**
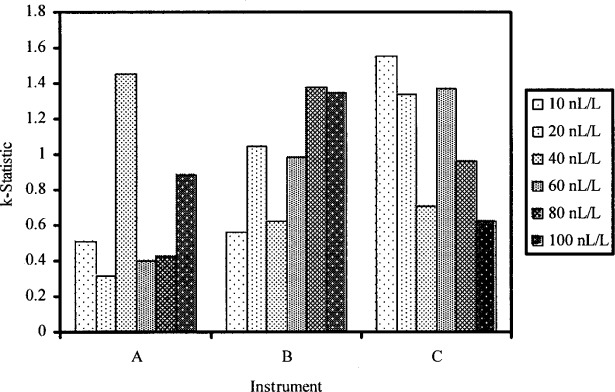
Chart of *k*-statistic

**Fig. 3:**
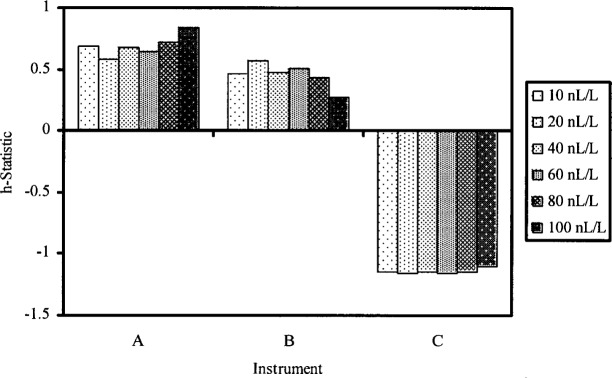
Chart of *h*-statistic

The objectives of the first part of data analysis are as follows. (1) Check for evidence of instrument effects. (2) Check whether within-instrument standard deviations are similar. (3) Check that there are no highly discrepant instrument arithmetic means. The four measurements for a given nominal level and instrument are referred to as a cell. The data is investigated by examining the *k*-statistic and the *h*-statistic for each cell. Formulas for the *k*-statistic and *h*-statistic are given in Appendix A. The *k*-statistic is normalized within-instrument standard deviation. It is used to check whether within-instrument standard deviations are similar. The *h*-statistic is standardized cell arithmetic mean. It is used to check whether any cell arithmetic means are highly discrepant. Computed values of *k*-statistic and *h*-statistic are presented in Table 2 and charted in Figs. 2 and 3, respectively. The first impression from the chart of *k*-statistic is that the within-instrument standard deviations are different. However, within-instrument standard deviations are based on only four measurements, so we can expect large random fluctuations among them even when there is no instrument or level effect. In view of the small number of measurements, we conclude from the chart of *k*-statistic that within-instrument standard deviations are not widely different. This conclusion is supported by the statistical test discussed in Ref. [4], which is applicable when the data can be assumed to have normal distribution. Thus the repeatability standard deviation for a nominal concentration can be computed using all data for that level. The chart of *h*-statistic shows that there is a clear evidence of instrument effects and that there are no highly discrepant instrument arithmetic means. Thus the reproducibility standard deviation for a nominal concentration can be computed using all data for that level. In summary, the data are reasonably valid for quantifying repeatability and reproducibility standard deviations.

**Fig. 4:**
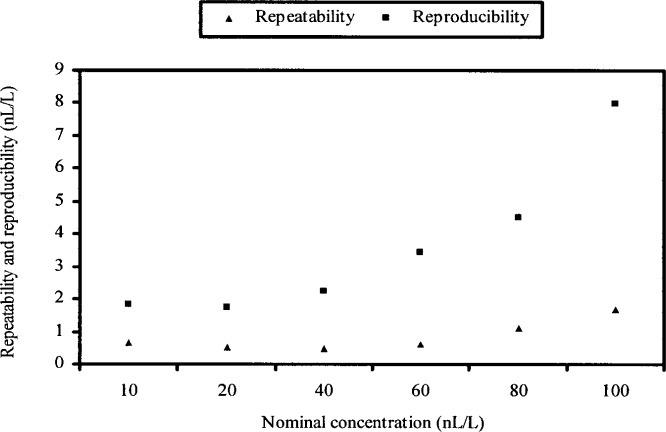
Chart of repeatability and reproducibility standard deviations.

Repeatability and reproducibility standard deviations are computed using the formulas given in the ASTM standard E691-99 and reproduced here in the Appendix. The computed values of the repeatability standard deviation, denoted by *s*_r_, and the reproducibility standard deviation, denoted by *s*_R_, are given in Table 3 and charted in Fig. 4. Since the reproducibility standard deviation includes the repeatability standard deviation, it is always larger. Both the repeatability standard deviation *s*_r_ and the reproducibility standard deviation *s*_R_ tend to increase with the nominal level. This is to be expected and is consistent with previous findings about the effect of flow-rate [2]. A quadratic polynomial (not shown here) fits well the charts of repeatability standard deviation *s*_r_ and reproducibility standard deviation *s*_R_ versus the level of nominal concentration. Table 3 also includes arithmetic means of the difference (δ*x*) for different nominal concentrations. The arithmetic means are small relative to their reproducibility standard deviations. From Fig. 1, we note that the negative values of the arithmetic mean of the difference (δ*x*) are largely caused by data from the PTMG labeled C.

## 3. Conclusion

The graphical investigation of data using the *k*-statistic and the *h*-statistic shows that they are reasonable to quantify repeatability and reproducibility standard deviations in PTMG measurements. Thus the repeatability and reproducibility standard deviations given in Table 3 should be of interest to the scientific and technical community. Repeatability standard deviation ranges from 1 nL/L to 2 nL/L, approximately. Reproducibility standard deviation ranges from 2 nL/L to 8 nL/L approximately. These standard deviations quantify possible variation in measurement of water vapor concentration generated by typical PTMG instruments used in the semiconductor industry.

## Figures and Tables

**Table 1:** Data on difference in measurement: calculated value minus measured value

Nominal concentration (nL/L)	Test instrument	Test 1 (nL/L)	Test 2 (nL/L)	Test 3 (nL/L)	Test 4 (nL/L)
10	A	1.07	1.42	1.07	0.62
10	B	0.79	0.81	0.10	0.88
10	C	−3.26	−2.20	−2.43	−0.83
20	A	0.37	−0.04	0.17	0.23
20	B	0.75	0.26	0.26	−0.61
20	C	−2.57	−3.06	−1.78	−3.46
40	A	0.57	0.45	−0.34	1.39
40	B	0.32	0.23	−0.36	0.16
40	C	−3.59	−3.17	−3.19	−3.89
60	A	0.87	0.58	0.86	1.19
60	B	0.41	0.64	0.94	−0.49
60	C	−4.89	−6.50	−4.74	−4.76
80	A	0.76	0.35	1.45	1.06
80	B	−2.12	−0.48	1.60	−0.49
80	C	−7.33	−8.76	−6.83	−6.28
100	A	5.43	3.95	2.50	5.73
100	B	−2.44	2.33	−1.37	1.44
100	C	−11.91	−11.50	−9.93	−9.89

**Table 2:** Table of *k*-statistic and *h*-statistic

Nominal concentration (nL/L)	Test instrument	*k*-statistic	*h*-statistic
10	A	0.506	0.687
10	B	0.564	0.460
10	C	1.557	−1.147
20	A	0.316	0.583
20	B	1.049	0.572
20	C	1.341	−1.155
40	A	1.451	0.673
40	B	0.626	0.476
40	C	0.709	−1.149
60	A	0.399	0.650
60	B	0.986	0.502
60	C	1.367	−1.152
80	A	0.421	0.716
80	B	1.378	0.427
80	C	0.961	−1.143
100	A	0.888	0.836
100	B	1.349	0.272
100	C	0.626	−1.108

**Table 3:** Repeatability standard deviation, reproducibility standard deviation, and arithmetic mean of difference in measurement for six levels of nominal concentrations

Nominal concentration (nL/L)	Repeatability *s*_r_ (nL/L)	Reproducibility *s*_R_ (nL/L)	Arithmetic mean (nL/L)
10	0.65	1.85	−0.16
20	0.54	1.73	−0.79
40	0.49	2.22	−0.95
60	0.62	3.43	−1.32
80	1.11	4.52	−2.26
100	1.68	7.96	−2.14

## 4. Appendix A. Formulas

The following formulas are defined for a fixed level of the nominal moisture concentration. Let *x_ij_* denote the *j*-th measurement for the *i*-th apparatus, where *j* = 1, 2,…, *J* and *i* = 1, 2,…, *I*. Here *J* = 4 and *I* = 3. Let *x_i_* be the arithmetic mean and *s_i_* the sample standard deviation of the *J* measurements. The *k*-statistic is the square-root of normalized variance $s_{i}^{2}/\left(\Sigma_{i}s_{i}^{2}/I\right)$ for *i* = 1, 2,…, *I*. It is used to compare the variation of within-instrument standard deviations. Let *x* be the arithmetic mean and *s* be the standard deviation of the arithmetic means *x*_1_,…, *x_I_*. The *h*-statistic is the standardized instrument mean (*x_i_* – *x*)/*s* for *i* = 1, 2,…, *I*. Both the *k*-statistic and *h*-statistic are dimensionless quantities. The repeatability standard deviation is defined as $s_{r}=\sqrt{\Sigma_{i}s_{i}^{2}/I)}$. This is the denominator of the *k*-statistic. The reproducibility standard deviation is defined as $s_{R}=\max\{s_{r},\sqrt{\left[s^{2}+(1-1/J)s_{r}^{2}]\right\}}$, Ref. [4].
